# A rare case report: acute necrotizing encephalopathy and acute fulminant myocarditis

**DOI:** 10.3389/fcvm.2025.1574397

**Published:** 2025-05-27

**Authors:** Juan Ma, Chenliang Pan, Nan Bai, Shanshan Zhang, Peiling Mi, Yongling Wa, Andong Lu

**Affiliations:** Department of Cardiovascular Medicine, The First Hospital of Lanzhou University, Lanzhou City, Gansu, China

**Keywords:** acute necrotizing encephalopathy, acute fulminant myocarditis, shock, IL-6, tocilizumab (IL-6 inhibitor)

## Abstract

**Background:**

Acute necrotizing encephalopathy (ANE) is a rare condition characterized by multiple symmetrical brain lesions mainly involving the thalamus. Acute fulminant myocarditis is a diffuse inflammatory disease of the myocardium characterized by acute onset, rapid progression, and a high risk of death. Its pathogenesis involves excessive activation of the innate immune system and the formation of an inflammatory storm. Both conditions are thought to be caused by viral infections. We present a case of ANE with fulminant myocarditis. Reporting this case is important due to the rarity and the critical interplay of these two severe conditions occurring simultaneously.

**Case presentation:**

A 16-year-old student presented with a 3-day history of high fever, cough, and expectoration, followed by multiple episodes of convulsive seizures. Despite high doses of vasoactive medications, the patient exhibited low blood pressure and elevated lactate levels. Portable echocardiography revealed diffuse decreased left ventricular motion with severe left ventricular dysfunction (ejection fraction < 30% by visual estimation). The patient was diagnosed with acute fulminant myocarditis. The patient remained comatose with a Glasgow coma scale (GCS) score of 3 (E1VeM1). Brain CT and MRI revealed bilateral striatal, thalamic, and brainstem lesions, typical of ANE. Consequently, a diagnosis of ANE accompanied by fulminant myocarditis was considered. The treatment regimen included high doses of glucocorticoids, immunoglobulins, tocilizumab, and V-A ECMO (Veno-arterial extracorporeal membrane oxygenation) life support. The patient showed significant recovery of cardiac function and was discharged after approximately 24 days of rehabilitation.

**Conclusion:**

This case report highlights the coexistence of ANE and fulminant myocarditis. The underlying mechanisms remain unclear. Early recognition of these two conditions is crucial for prognosis, though challenging. This report underscores the need for heightened awareness and prompt, comprehensive treatment strategies to improve outcomes in such complex cases.

## Background

Acute necrotizing encephalopathy (ANE) is a rare, acute, life-threatening condition characterized by symmetrical brain lesions affecting the thalami, putamina, cerebral and cerebellar white matter, and brain stem tegmentum following various viral illnesses (1, 2). Fulminant myocarditis (FM) is a rapid progressing, acute diffuse inflammatory disease of the myocardium with a high risk of death (3). Infection is the most common cause of the diseases, and viruses are the predominant pathogens (2, 4). Here, we report a case of ANE accompanied by fulminant myocarditis. To the best of our knowledge, this is the rare reported case of coexisting FM and ANE.

## Case presentation

A 16-year-old student presented to the emergency department of a local hospital with a 3-day history of high-grade fever, cough, and expectoration, followed by multiple episodes of convulsive seizures. The patient had no underlying disease, significant family history, or current medications. Clinical examination revealed a comatose patient with a Glasgow Coma Scale (GCS) score of 6/15. She was intubated due to the low GCS and transferred to the intensive care unit. Simultaneously, the patient experienced a rapid decrease in blood pressure and an increase in lactate levels, and blood pressure as low as 80/50 mmHg and lactate peaking at 10.8 mmol/L. Despite high doses of vasoactive medications, she maintained low blood pressure and elevated lactate levels. The doses of norepinephrine and dopamine administered were 1.5 ug/kg/min and 10 ug/kg/min, respectively. Consequently, her physician contacted us for extracorporeal membrane oxygenation (ECMO). The patient was then transported to our hospital on venoarterial (V-A) ECMO.

Portable echocardiography revealed diffuse decreased left ventricular motion with severe left ventricular dysfunction [ejection fraction (EF) <30% by visual estimation]. Laboratory findings showed marked elevated cardiac troponin I(TnI) (3.814 ng/ml, reference range 0–0.3 ng/ml), serum creatine phosphokinase isoenzyme (CK-MB) (26.54 ng/ml, reference range 0–6.36 ng/ml), N-terminal pro-brain type natriuretic peptide (>35,000 pg/ml, reference range 0–300 pg/ml), and D-dimer (>20 ug/ml, reference range 0–0.5 ug/ml). Mycoplasma pneumoniae IgM(MP-IgM) antibody was also positive. Additionally, the patient had acute liver failure, acute kidney injury, and coagulation dysfunction. Laboratory test results are presented in Table 1. And Time line was shown in Table 2.

**Table 1:** The laboratory test results.

Laboratory test	Laboratory test results			
	Before admission	Reference range	Day 1 post-admission	Reference range
TnI(ng/ml)	3.814	0–0.3	4.7	0.01–0.023
NT-proBNP(pg/ml)	>35,000	0–300	20,000	300–450
Leukocytes (10*10^9^/L)	10.82	4–10	6.53	3.5–9.5
Hemoglobin (g/L)	130	110–170	95	130–175
Platelets(10*10^9^/L)	75	100–300	37	125–350
CRP(mg/L)	ND	-	25.9	0–4
PCT(ng/ml)	9.76	0–0.5	6.39	0–0.046
Il-6(pg/ml)	131	0–1.5	21.9	0–7
ALT(U/L)	2,234	0–50	3,219	9–50
AST(U/L)	5,779	0–38	1,532	15–40
Albumin(g/L)	31.8	35.3–52.9	25.3	40–55
BUN(mmol/L)	10.9	1.7–8.3	8.99	3.1–8.0
Cr(mmol/L)	129	35–80	104.4	57–97
NA + (mmol/L)	148	135–144	144.8	137–147
LDH(U/L)	5,851	0–65	3,315	120–250
Glucose(mmol/L)	6	3.9–5.8	8.1	3.9–6.1
PT(s)	32	9–14	40.3	9.4–12.5
Fibrinogen(g/L)	0.346	0–5	0.4	2.39–4.98
D-dimer(ug/ml)	193	0–0.5	253	0–0.5
Lactate(mmol/L)	9.58	0.5–1.6	10.8	0.5–1.6

TnI, troponin I; NT-pro-BNP, N-terminal pro-brain natriuretic peptide; CRP, C-reactive protein; PCT, procalcitonin; ND, not performed; BUN, blood urea nitrogen; Cr, creatinine; LDH, lactate dehydrogenase; PT, prothrombin time.

**Table 2:** Time line.

Time line	Treatment	Condition
D1	NO	High fever, cough, and expectoration.
D2	Mechanical ventilation and Vasoactive drug support	Multiple episodes of convulsive seizures, GCS E1VeM1.
	ICU in the local hospital.	
D2	V-A ECMO.	Then the patient went into shock, and bood pressure as low as 80/50 mmHg and lactate peaking at 10.8 mmol/L.
		Cardiac function deterioration
D3	ECMO	The patient was then transported to our hospital on V-A ECMO.
	Ventilatory	
	Azithromycin Methylprednisolone	
	Immunoglobulin	
D6	Stop using sedatives and analgesics	Remained in a coma(E1VeM1)
D9	ECMO was removed	Remained in a coma(E1VeM1)
D12	Tocilizumab	CT revealed lesions involving the bilateral striatum, thalamus, and brainstem, typical for ANE.
D13	Ehabilitation	Responding to painful stimuli
D16	Ehabilitation	Transported to the ehabilitation department
D40	Ehabilitation	Discharge
		Walk and speak clearly

On admission, vital signs were as follows: temperature: 36.9°C, heart rate: 114 beats per minute (bpm), respiratory rate: 13 cycles per minute (cpm), blood pressure: 84/73 mmHg, oxygen saturation (SpO2): 98%. The GCS was graded 3 (E1VeM1). The pupils size was 2 mm, equal but unreactive to light.

The treatment regimen included ECMO support, ventilatory support, azithromycin anti-infection therapy, methylprednisolone(500 mg/day of methylprednisolone for 3 days), and immunoglobulin(2 g/kg total dose of IVIG over 5 days). To address elevated intracranial pressure (ICP), the patient received intravenous mannitol (20%, 125 ml every 6 h) with close monitoring of neurological status and electrolytes. The patient had repeated convulsions during the course of the disease, so we immediately gave antiepileptic drug (AED) which including intravenous sodium valproate (1 mg/kg/hour continuous intravenous pumping) for 5 days after admission. Following treatment, cardiac function recovered up to 38% of EF on the sixth day of ECMO therapy, and weaning from ECMO became possible 6 days after treatment initiation.

On the third day of admission, sedation and analgesia were discontinued, but the patient remained in a coma with a GCS score of 3(E1VeM1). Lumbar puncture revealed an opening pressure of 130 mmH2O. Cerebrospinal fluid (CSF) analysis revealed an elevated erythrocyte count of 20  ×  10^6^/L with a normal leukocyte count. CFS protein levels were elevated. CSF glucose, and chloride levels were normal, and the culture and cytology of the CSF were negative. Laboratory test results are presented in Table 3.

**Table 3:** Relevant serum and CSF studies in our patient.

Results of relevant serum and CSF studies				
Infectious		Immunologic	Results	Reference range
HBV	Negative	Antinuclear antibody	Negative	
HIV	Negative	C3 Complement(g/L)	0.203	0.7–104
Syphilis	Negative	C4 Complement(g/L)	0.09	0.1–0.04
EBV IgG/DNA	Negative	IgG(g/L)	8.11	8.6–17.4
CMV DNA	Negative	IgM(g/L)	0.88	0.5–2.8
SRSA-COV-2 PCR	Negative	IgA(g/L)	1.65	1.0–4.2
Respiratory Viral PCR^a^	Negative			
Respiratory Viral IgM^b^	Negative			
Mycoplasma Pneumonia IgG/M	positive/weakly Positive			
Genetics	RANBP2	Negative		
CSF	Basic	Infectious		
Glucose (mmol/L)	4.31(Reference range: 2.5–4.5)	Bacterial and fungal culture	Negative	
Protein (g/L)	0.81(Reference range: 0.15–0.45)	Next Generation Sequencing	Negative	
RBC (10*10^9^/L)	25(Reference range: 0)	Ink stain	Negative	
WBC (10*10^9^/L)	2(Reference range: 0–8)	Acid-fast stain	Negative	

HBV, hepatitis B virus; HIV, human immunodeficiency virus; EBV, Epstein–Barr virus; IgG, immunoglobulin G; CMV, cytomegalovirus; IgM, immunoglobulin M; SARS-COV-2, severe acute respiratory syndrome coronavirus disease 2019; IgA, immunoglobulin A; PCR, polymerase chain reaction; IgE, immunoglobulin E; DNA, deoxyribonucleic acid; WNL, within normal limits; RANBP2, RAN-binding protein 2.

^a^ Adenovirus, influenza A&B, respiratory syncytial virus (RSV), and Mycoplasma pneumoniae.

^b^ Echovirus, Coxsackievirus, adenovirus, respiratory syncytial virus (RSV), parainfluenza virus-1,2,3, and Chlamydia pneumoniae.

**Figure 1:**
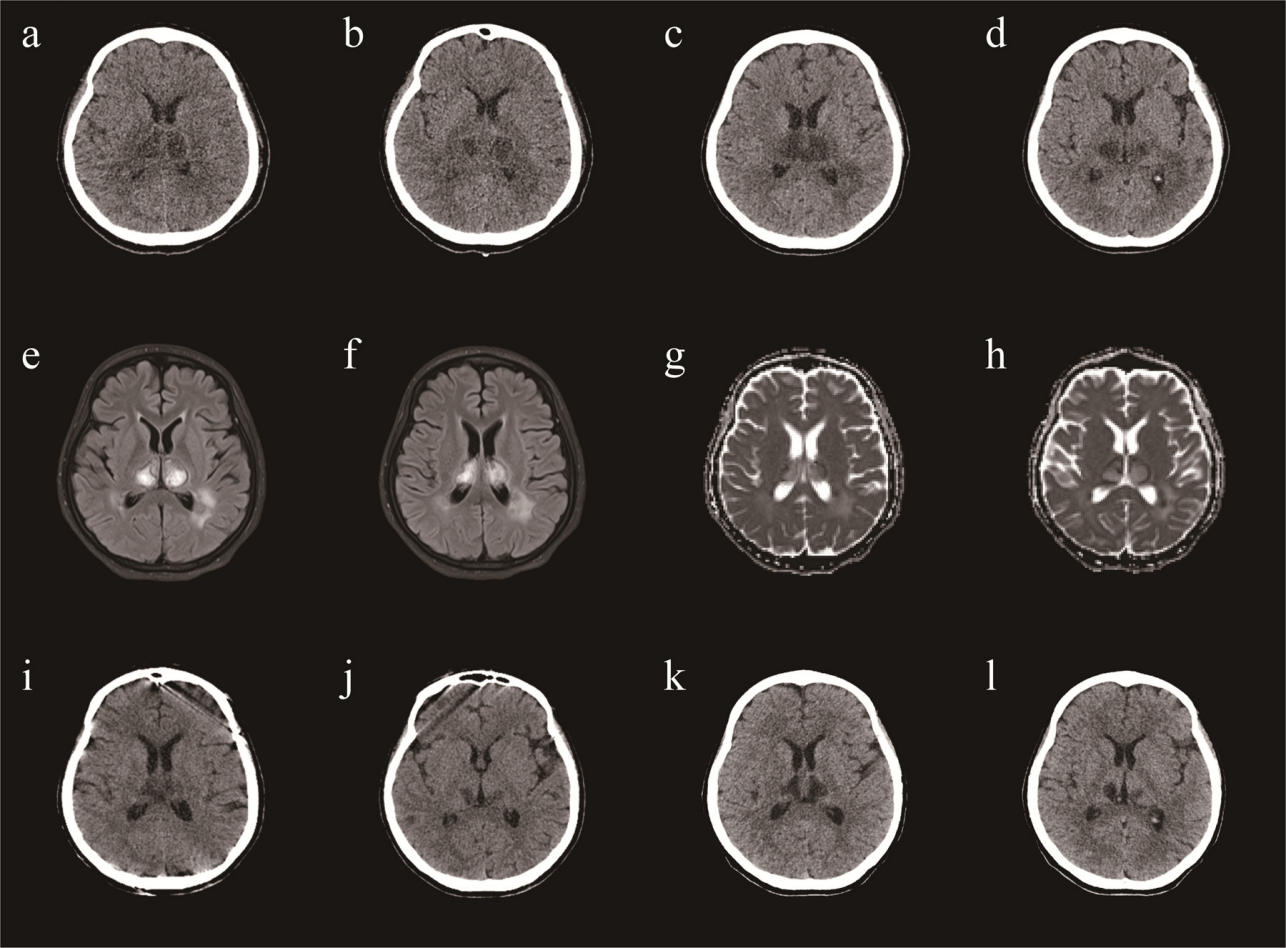
A brain CT **(a,b)** demonstrating symmetric lesions involving thalami, striatum and brain stem, consistent with acute necrotizing encephalopathy in day 12. A repeat CT revealed that the lesions in the head had reduced **(c,d)** in day 20. A repeat CT revealed that the lesions in the head had reduced **(e,f)** in day 38. One month after discharge, a repeat CT revealed that the lesions in the head had reduced **(g,h)**. MRI **(i,j)** demonstrating symmetric lesions involving thalami, striatum and brain stem, consistent with acute necrotizing encephalopathy in day 22.

Brain Computed Tomography (CT) and Magnetic Resonance Imaging (MRI) revealed lesions involving the bilateral striatum, thalamus, and brainstem, typical for ANE (Figures 1A,B,I–L). A diagnosis of ANE accompanied by fulminant myocarditis was considered. Therefore, tocilizumab was included in the treatment regimen. Tocilizumab was used in hospital day 9 (8 mg/hg of tocilizumab for 1day). On the tenth day of admission, the patient began to regain consciousness. She was transferred to a general ward after 13 days. Echocardiography showed a significant improvement in left ventricular function (EF = 61%). The patient was discharged after approximately 24 days of rehabilitation. At the time of discharge, the patient could walk and speak clearly. She was discharged with a prescription for cardioprotective agents, including metoprolol, sacubitril/valsartan, spironolactone, furosemide, and dapagliflozin. One month after discharge, the patient was able to walk on her own, her speech was cleared, there were no remaining symptoms, and her heart function was normal. A repeat CT revealed that the lesions in the head had reduced (Figures 1G,H).

## Discussion

ANE typically arises secondary to neurological complications of influenza or other viral infections (1, 5, 6). The disease progresses rapidly and often results in severe disability or death. Characteristic neuro-radiologic findings include multifocal and symmetric lesions, mainly affecting the bilateral thalami, cerebral periventricular white matter, brainstem tegmentum, and cerebellum (1, 7). Treatment of ANE is mainly supportive, with early high-dose intravenous steroids (methylprednisolone) leading to better outcomes. Tocilizumab may also be beneficial in improving morbidity outcomes (5, 8–10). Additionally, plasma exchange may be associated with increased survival in patients with ANE (10, 11).

FM is mainly caused by a variety of viral infections and is characterized by the rapid onset of cardiogenic shock (3, 12–14). In this case, the patient exhibited features such as sudden onset, rapid emergence of severe hemodynamic disorders, severe myocardial injury, and diffuse decreased ventricular wall movement, aligning with the diagnostic criteria of fulminant myocarditis (3). Active comprehensive treatment should be initiated as soon as possible for fulminant myocarditis, including anti-infection, antiviral therapy, glucocorticoids, gamma globulin, life support measures, and general treatment to nourish the myocardium and reduce cardiac load, etc (3, 4, 15, 16).

This is the rare case of ANE and FM coexisting in the same patient. ANE is most often seen following viral infection, and common viruses include influenza virus, SARS-CoV-2 and so on (2, 8, 10, 17). About FM, viral infection is also considered a major cause of myocarditis, including Coxsackie B virus, adenovirus and so on (4). The pathogenetic testing was positive for MP antibodies in this case, whereas all other pathogenetic tests yielded negative results. According to previous literatures, we found that MP can also cause ANE or FM (18, 19). MP-IgM can be detected within 1 week after clinical onset, and peaked at 3 weeks (20, 21). MP-IgG antibody can be detected about 2 weeks after infection, peaked at 5 weeks and maintained for a long time (20, 21). The median duration of persistence of MP DNA in the body is 7 weeks after infection, and the period cannot be shortened even if adequate antibiotic treatment is given (22). However, PCR-positive patients at presentation are far less than single IgM-positive patients, and PCR-negative patients, especially lately presented, are common over 20%–50% of study subjects (23). In this patient, the MP-IgM in the local hospital was positive, while the IgM and IgG antibodies were positive in our hospital, although the PCR was negative. Therefore, MP was considered to be the cause of the case.

It is believed that the cytokine storm caused by excessive innate immune activation caused by various causes and rapid triggering of immune cells to release a large number of inflammatory factors, also known as “inflammatory storm”, is an important reason for FM (3, 12). The “inflammatory storm” formed by a large number of cytokines and inflammatory mediators, including soluble growth stimulating expression gene 2 protein (sST2),interleukin-1 (IL-1), IL-6 and tumor necrosis factor (TNF)-*α* among others, may be the main cause of severe myocardial injury and pump failure (4). The most common hypothesis for the pathogenesis of ANE is also a “cytokine storm” involving interleukin-6 (IL-6), tumor necrosis factor-alpha (TNF-α), IL-10, IL-15 among others (5). IL-6 palys a import role in the pathogenesis of ANE and higher IL-6 levels correlate with more severe disease. Therefore, the causes of the two diseases may be similar and the treatment of them include glucocorticoids, immunoglobulin and so on. IL-6 inhibition is a natural choice when considering a more targeted approach to treating ANE and FM. At present, some studies suggest that tocilizumab can be used in the treatment of FM (24, 25). The proportion of patients who was ANE found to have a good outcome after receiving tocilizumab was much higher than those not receiving tocilizumab (17). IL-6 receptor blockade exerts cardiac beneficial effects by antiviral and immunomodulatory actions after induction of an acute murine CVB3 virus myocarditis (26). For this patient, we administered high doses of glucocorticoids, immunoglobulins, tocilizumab, and V-A-ECMO life support. Laboratory tests showed positive mycoplasma antibodies, and azithromycin was administered to treat the infection.

In conclusion, we report a rare case of ANE associated with FM. Physicians should be aware of this uncommon but life-threatening complication when patients with fulminant myocarditis develop consciousness disturbances.

## Data Availability

The original contributions presented in the study are included in the article/Supplementary Material, further inquiries can be directed to the corresponding author.
